# Vascular Steal in White Matter of Non-Flow-Limited Cerebral Hemispheres Following Acetazolamide Challenge Using Arterial Spin Labeling Magnetic Resonance Imaging

**DOI:** 10.3390/brainsci16020160

**Published:** 2026-01-30

**Authors:** Rahim Ismail, Denes Szekeres, Stephen Smith, Giovanni Schifitto, Timothy Hoang, Evan McConnell, Matthew Bender, Henry Wang

**Affiliations:** 1 Department of Radiology, University of Texas Southwestern Medical Center, Dallas, TX 75390, USA; 2 Department of Imaging Sciences, University of Rochester Medical Center, Rochester, NY 14642, USA; 3 Department of Neurology, University of Rochester Medical Center, Rochester, NY 14642, USA; 4 Department of Neurological Surgery, University of Rochester Medical Center, Rochester, NY 14642, USA

**Keywords:** vascular steal, vascular reserve, arterial spin labeling, leukoaraiosis, acetazolamide, cerebral vaso-occlusive disease

## Abstract

**Background**: Vascular disease is a known risk factor for the development of leukoaraiosis. Assessment of cerebral blood flow (CBF) was performed at baseline and after acetazolamide (AZM) challenge to evaluate for vascular reserve and steal within the brain. Little has been reported on the physiological reserve in the non-flow-limited hemispheres. This study attempts to evaluate for vascular steal in areas commonly involved in leukoaraiosis, in the setting of pharmaceutically induced states of increased CBF. **Methods**: Patients who underwent AZM challenge MRI from 2014 to 2021 and a cerebral angiogram within one year were included. Patients with bilateral disease or non-diagnostic imaging artifacts were excluded. MRIs were obtained after 1 g of AZM was administered 5 and 10 min prior to acquisition. Augmentation and steal maps were generated. Regression analysis, Pearson correlation coefficient, two-sample *t*-test, Spearman and Mann–Whitney U analyses were utilized for statistical evaluation. **Results**: A total of 38 patients with unilateral cerebral vaso-occlusive disease underwent the AZM challenge. Vascular steal and T2 hyperintensities were assessed in non-flow-limited hemispheres (NFLH) and flow-limited hemispheres (FLH). A moderate correlation was demonstrated between NFLH steal and NFLH T2 hyperintensities (rs = 0.48, *p* = 0.0020). A weak correlation without statistical significance was demonstrated between ipsilateral T2 and contralateral T2 hyperintensities (rs = 0.27, *p* = 0.10). **Conclusions**: The vascular steal phenomenon was demonstrated in the distal cerebral vasculature of cerebral white matter even in the absence of upstream flow-limiting stenosis, suggesting an inherent vulnerability of these structures to hemodynamic fluctuations and possiblly contributing etiology to leukoaraiosis.

## 1. Introduction

Acetazolamide challenge MRI is a well-established means of assessing autoregulatory vasodilation in steno-occlusive cerebrovascular disease [1]. This is accomplished by obtaining baseline measurements of cerebral blood flow, followed by measurements after augmentation with acetazolamide. Typically, 1 g is given 10–15 min prior to the acquisition of augmented images [2,3]. The most common indication for this study is to assess the distal cerebrovascular reserve in patients with steno-occlusive disease.

In patients with chronic unilateral cerebrovascular disease, acetazolamide challenge in the non-diseased contralateral hemisphere allows for a unique evaluation of cerebrovascular reserve and relational hemodynamics. At our institution, this study is readily performed in patients with chronic cervical carotid, intracranial carotid or proximal MCA steno-occlusive disease for risk stratification and surgical planning. The aim of this study was to evaluate the patterns of steal seen in the incidentally captured NFLH to better understand the pathophysiology of cerebral small vessel vascular disease. Our hypothesis is that vascular steal from deep white matter perfusion (e.g., centrum semiovale) is a common physiologic mechanism for maintaining perfusion in more critical structures and results in transient ischemia, contributing to T2 hyperintensities commonly seen on brain MRIs.

## 2. Materials and Methods

### 2.1. Patient Selection

This retrospective study was written according to the Strengthening the Reporting of Observational Studies in Epidemiology (STROBE) guidelines [4]. All patients who underwent an acetazolamide challenge at our institution from 2014 to 2022 were initially included in this study. An image acquisition or individual series were excluded if felt to be non-diagnostic secondary to significant artifacts such as patient movement. Each patient’s most recent cerebral angiogram report within one year of MRI acetazolamide challenge was reviewed and screened for contralateral disease. Any description of bilateral disease was flagged and excluded if found to be flow-limiting by a board-certified neuroradiologist with more than 25 years of experience. Data for participants in this retrospective review were obtained as part of a larger retrospective review of acetazolamide MRI challenge and approval was obtained from our institution’s Institutional Review Board. Clinical data such as patient age, diabetes status, sex, smoking history, total cholesterol, HDL, systolic blood pressure, hypertension treatment, and race were acquired from the electronic medical record. Data was acquired as close as possible to the date of MRI. If multiple entries were entered for the same day, the entry most consistent with the patient’s prior clinical history was chosen.

### 2.2. Imaging Acquisition and Evaluation

Our group has established an easily implemented protocol to assess for cerebrovascular reserve (CVR) in patients. A standard 1 g of acetazolamide (AZM) was administered to all patients 5 and 10 min prior to image acquisition. Routine sequences such as FLAIR and DWI were performed prior to the acquisition of pre-AZM ASL images. Our group’s ASL scanning protocol is performed on a 3T GE Discovery 750w scanner (General Electric, Milwaukee, WI, USA) equipped with an 8-channel receiver head coil and includes a pCASL spiral 3D acquisition with 8 arms with 512 points, TR = 4830 ms, TE = 10.7 ms, BW = 62.5 kHz, PLD = 2025 ms, slice thickness = 4.0 mm (~34 slices), FOV = 24 × 24 cm, giving an effective resolution of 3.73 mm and a total scan time of 4.40 min. Following the collection of pre-AZM ASL images, 1000 mg of AZM was given over the course of 2 min. The ASL sequence was collected at 5 and 10 min following the initial injection of AZM. This redundancy allows for greater confidence in a sequence that has an inherently low signal-to-noise ratio compared to other more well-established MR perfusion techniques and the ability to reattempt data acquisition in the event of technical challenges. Also, if early termination of the protocol is required due to anxiety or urinary urgency, as AZM is a well-known diuretic, a 5 min ASL scan has been obtained, which may be diagnostic. Once pre- and post-AZM (5 and 10 min) time points have been collected, augmentation and steal maps can be generated using simple arithmetics. To generate an augmentation map, the pre-AZM CBF images are subtracted from the post-AZM CBF images. This gives important information on which areas have or lack appropriate CVR. In addition, post-AZM CBF images were subtracted from the pre-AZM CBF images to generate a steal map to assess for areas where CBF decreases in response to AZM challenge.

Data with significant artifacts, such as motion, were excluded if felt to be unreliable for analysis. Areas of steal and areas of T2 hyperintensities in the deep white matter were categorized by a radiologist as Grade 0—absent, Grade 1—punctate foci, Grade 2—beginning confluence or Grade 3—large confluent areas in a fashion based on the Fazekas scale for white matter lesions [5].

Areas of T2 hyperintensities in the deep white matter were categorized by a radiologist as Grade 0—absent, Grade 1—punctate foci, Grade 2—beginning confluence or Grade 3—large confluent areas in a fashion based on the Fazekas scale for white matter lesions [5]. Areas of steal were graded similarly to the Fazekas scale.

### 2.3. Statistics

Statistical analysis was performed using Microsoft Excel and the Data Analysis ToolPack add-in as well as GNU PSPP (Version 1.6.2-g78a33a). Regression analysis using the Pearson correlation coefficient, as well as the *t*-test: Two-Sample Assuming Unequal Variances were utilized. Spearman correlation was utilized to quantify the relationship between the ordinal data sets. The Mann–Whitney U test was utilized to quantify the relationship between the nominal and ordinal data sets. For all statistical tests, a *p*-value of <0.05 was used to indicate statistical significance.

## 3. Results

### 3.1. Patient Demographics

Demographics of patients included in the study are provided in Table 1. A total of 40 patients with diagnoses of unilateral chronic steno-occlusive disease underwent acetazolamide challenge MRIs between 2014 and 2022. Further screening of radiology reports and images revealed two patients to have flow-limiting stenosis bilaterally, and these patients were excluded. Subsequently, 38 different patients received the acetazolamide challenge with 5 and 10 min acquisitions. A total of 22 male and 16 female patients were evaluated. A total of 11 patients had cervical carotid stenosis, 15 patients had middle cerebral artery stenosis, and 12 patients had intracranial carotid stenosis. A total of 16 patients had left-sided stenosis, and 22 patients had right-sided stenosis. The findings are summarized in Table 1 and Table 2 below.

### 3.2. Analysis of Non-Flow-Limited Hemispheres

10 patients had Grade 0 disease in the centrum semiovale. 20 patients had punctate foci areas of T2 hyperintensities in the centrum semiovale. 8 patients had beginning of confluent areas of T2 hyperintensities in the centrum semiovale. 0 patients had large confluent areas of T2 hyperintensities in the centrum semiovale. On steal subtraction maps, 4 patients had no areas of steal, 15 patients had punctate foci areas of steal in the centrum semiovale, 18 patients had the beginnings of confluent areas of steal and 1 patient had large confluent areas of steal in the centrum semiovale. The findings are summarized in Table 3.

### 3.3. Analysis of Flow-Limited Hemispheres

A total of two patients had no areas of T2 hyperintensities in the centrum semiovale. A total of six patients had punctate foci areas of T2 hyperintensities in the centrum semiovale. A total of 15 patients had the beginnings of confluent areas of T2 hyperintensities in the centrum semiovale. A total of 15 patients had large confluent areas of T2 hyperintensities in the centrum semiovale. On steal subtraction maps, 1 patient had no areas of steal, 4 patients had punctate foci of steal, 26 patients had the beginnings of confluent areas of steal, and 7 patients had large confluent areas of steal in the centrum semiovale. The findings are summarized in Table 3.

### 3.4. Statistical Analysis

#### 3.4.1. Correlation of NFLH Steal and NFLH T2 Hyperintensities

Results demonstrated a moderate correlation of (rs = 0.48) and a high level of statistical significance (*p* = 0.0020) (Table 4).

#### 3.4.2. Correlation of Ipsilateral T2 Hyperintensities and Contralateral T2 Hyperintensities

A weak correlation was determined (rs = 0.27); however, it was not found to be statistically significant (*p* = 0.10) (Table 4).

#### 3.4.3. Severity of Steal and Occlusion Location of Stenosis

No significant correlation was found between the location of the stenosis (i.e., cervical ICA vs. intracranial ICA terminus vs. MCA) and the degree of steal or T2 hyperintensity in either hemisphere (Table 5).

#### 3.4.4. Correlation of Steal and T2 Hyperintensities with ASCVD Score

No significant correlation was found between either ipsilateral or contralateral steal or T2 hyperintensities and ASCVD score. (Table 6).

## 4. Discussion

**Figure 1:**
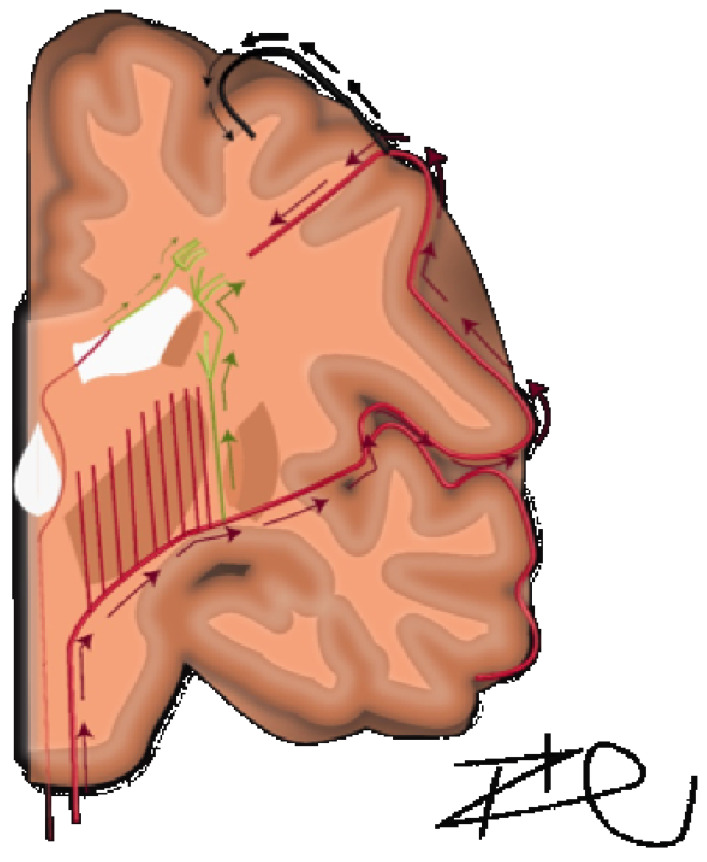
Arterial supply of white matter structures. Illustration demonstrating the arterial vascular supply to periventricular and deep white matter structures along with ventrofugal or ventropedial directionality: periventricular from choroidal (small green arrows), periventricular from terminal lenticulostriate (large green arrows), centripedal from cortical MCA branches (red arrows), and secondary vascular supply (black arrows) to the cortical u-fibers.

A detailed understanding of the centrum semiovale requires a brief review of its anatomical makeup, which was comprehensively described by Bogousslavsky and Regl in 1992 [6]. They discussed four different vascular territories: (1) the territory of the deep perforators (lateral and medial lenticulostriate, anterior choroidal, Hubner, anterior lenticulostriate and thalamostriate), (2) perforating branches of the superficial branches of the MCA, (3) junctional territories between deep and superficial territories of MCA, or (4) combined or extended subcortical infarcts. In terms of volume supplied, the predominant territory was the medullary arteries of the middle cerebral artery. The course of these arteries are relatively long and serpiginous and have been further detailed by Van den Bergh [7,8]. The middle cerebral medullary arteries must first reach the pial surface before beginning a 90-degree centripetal turn towards the lateral ventricles, spanning approximately 2–5 cm [9]. This is exaggerated in cases of chronic hypertension, in which vasculature demonstrates increased tortuosity [9]. There is a characteristic lack of arborization in centripetal, pre-periventricular arteries and thus function as terminal vessels without the contingency of collateral supply in times of decreased perfusion [10]. In contrast, subcortical U-fibers have dual supply with more superficial arterial vasculature and are characteristically spared from these white matter lesions in leukoaraiosis. Distinctly, in the immediate periventricular space, periventricular white matter is supplied by branches of the choroidal arteries and distal terminal lenticulostriate vessels traveling ventriculofugally (Figure 1) [8,10,11]. In the context of this anatomical makeup, there is a physiological mechanism implied for the lack of cerebrovascular reserve in the centrum semiovale.

Our data demonstrated that in cerebral hemispheres without significant flow limitations (non-flow-limited hemispheres or NFLH), increased physiological demand from the acetazolamide challenge resulted in a physiological steal phenomenon, away from white matter structures of the centrum semiovale. These findings suggest the presence of an intrinsically vulnerable cerebrovascular reserve in this region, even without proximal flow-limiting disease. Furthermore, the severity of steal in the NFLH demonstrated a strong relationship with the degree of T2 hyperintensities in the NFLH.

**Figure 2:**
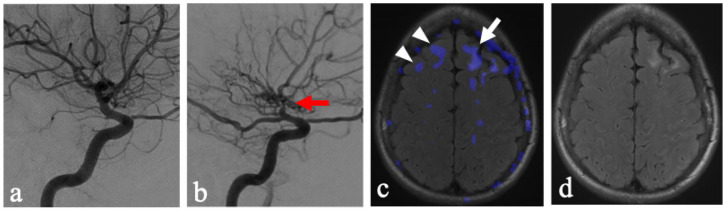
Adult patients with unilateral vaso-occlusive disease of the left anterior circulation. (**a**) A right-sided internal carotid artery DSA corresponding to the NFLH. (**b**) A left-sided internal carotid artery DSA corresponding to the FLH as evidenced by severe supraclinoid internal carotid artery stenosis (red arrow). (**c**) Axial T2/FLAIR MRI at the level of the centrum semiovale with areas of steal phenomenon overlaid in blue, demonstrating the occurrence of steal within the FLH (white arrow) greater than NFLH (white arrowheads). The steal grades for the right and left hemisphere were 1 and 3, respectively. (**d**) Axial T2/FLAIR MRI at the level of the centrum semiovale with T2/FLAIR hyperintensities in the left frontal lobe subcortical white matter. The Fazekas for the right and left hemisphere were graded independently as 0 and 2.

The findings presented suggest that inherent physiological vulnerability may play a role in the ubiquity of leukoaraiosis in this region and demand ischemia to be a key factor in the formation of T2 hyperintensities. While chronic hypertension and microangiopathy are often discussed as the precursor vasculature changes within the deep white matter tracts that, in turn, cause decreased vascular reserve and areas of leukoaraiosis [12], the inherent dynamic physiological vulnerabilities of the vasculature supplying the centrum semiovale may play a key role. This is supported by findings of steal in the centrum semiovale in the side without flow-limiting stenosis (Figure 2). These findings suggest an inherent physiological vulnerability of these regions to possible fluctuations in hemodynamics.

There is a paucity of clinical research investigating the pathophysiology of cerebrovascular reserve within the centrum semiovale. Martson et al. investigated 85 normal subjects under acetazolamide-augmented MRI and found hemodynamic changes after acetazolamide augmentation were less prominent in places of white matter hyperintensities when compared to normal-appearing white matter and concluded “a change in the hemodynamic status is present within the WMH, making these areas more likely to be exposed to transient ischemia inducing myelin rarefaction” [13]. This was recently supported by research correlating areas of poor cerebrovascular reserve with increased white matter hyperintensities, lacunar infarctions, and microhemorrhages [14]. Further support for altered hemodynamics was found in a study of vasoreactivity in multiple sclerosis, in which the vascular territory supplying the centrum semiovale was relatively limited in its autoregulation compared to surrounding territories in both MS patients as well as the controls [15].

One reason for the decreased vascular reserve may lie in the differential vascular responses to perfusion pressures at different vascular calibers. The cross-sectional diameter of the medullary arteries supplying the white matter is typically consistent until it approaches the lateral ventricle, and ranges from 100 to 200 μm [10]. Various human and animal studies suggest that smaller arterioles have a significant role in vascular autoregulation but are primarily activated in intermediate-to-severe levels of hypoperfusion [16,17,18]. Thus, while territories with smaller diameter arterioles demonstrate profound dilatation during severe hypotensive episodes [19] and protect against areas of necrosis and cavitation, they may still be vulnerable to diffuse rarefaction from episodes of less severe ischemia.

Perhaps even more critical is the length of the arterioles that supply the centrum semiovale. These consist of long medullary penetrating arterioles arising from the proximal MCA and are the longest parenchymal arterioles in the brain. Due to their length, these vessels demonstrate significantly higher flow resistance [20]. Therefore, there may be baseline vasodilatation existing in the normal physiological state. Thus, increased demand in these vessels results in limited additional vasodilatation and cerebrovascular reserve. In contrast, the shorter, cortical arterioles may demonstrate less baseline resistance, increased vasodilatation on demand and greater cerebrovascular reserve. Animal studies performed by Symon et al. [21] demonstrated episodes of zero blood flow in white matter territories at times of reduced perfusion pressures with the preservation of flow in the gray matter at the same degree of perfusion. This led the authors to conclude that “white matter possesses a less sensitive and effective regulatory mechanism than that in gray matter”. Furthermore, a study of newborn dogs demonstrated the preferential preservation of CBF in the gray matter but not the periventricular and occipital white matter [22].

**Figure 3:**
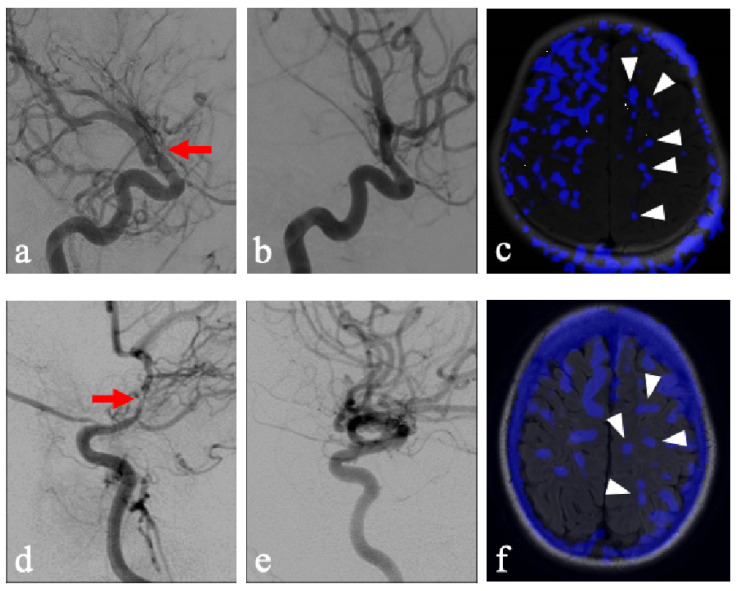
Two pediatric patients with unilateral vaso-occlusive disease of the right anterior circulation. (**a**) A right-sided ICA DSA with severe stenosis of the supraclinoid ICA (red arrow) corresponding to the FLH. (**b**) A left-side ICA DSA corresponding to the NFLH hemisphere. (**c**) Axial T2/FLAIR MRI with areas of steal phenomenon overlaid in blue, demonstrating the occurrence of steal within the NFLH (white arrowheads). The Fazekas for the right and left hemisphere were graded independently as 0 and 0. The steal for the right and left hemisphere were graded as 2 and 1, respectively. (**d**) A right-sided ICA DSA with severe stenosis of the supraclinoid ICA (red arrow) corresponding to the FLH. (**e**) A left-side ICA DSA corresponding to the NFLH hemisphere. (**f**) Axial T2 MRI with areas of steal phenomenon overlaid in blue, demonstrating the occurrence of steal within the NFLH (white arrowheads). The Fazekas for the right and left hemisphere were graded independently as 3 and 2, respectively. The steal for the right and left hemisphere were graded as 3 and 3, respectively.

A few considerations must be observed in the current methodology. Patients with chronic unilateral steno-occlusive disease may have developed compensatory contralateral contributions from the NFLH to the FLH via the Circle of Willis. Additionally, the NFLH in these patients may also demonstrate decreased overall reserve due to exposure to the same vascular risk factors which lead to disease on the FLH. Lastly, acetazolamide as a stressor may be considered too extreme of a vasodilator when compared to states of physiologically increased demand. Quantitative generalizability is therefore limited in this evaluation as various factors may exaggerate steal phenomenon in our patient population. However, while these may exaggerate the degree of steal in NFLH, it nonetheless uncovers a key compensatory mechanism inherent to areas that have demonstrated a propensity for leukoaraiosis. Particularly illustrative cases of this phenomenon include pediatric patients without documentation of chronic hypertension and who were unlikely to have long-term sequalae or compensation of hypertensive pathology, who were found to display this pattern of steal after acetazolamide administration (Figure 3). This study lays the groundwork for comparison with healthy controls, which would provide more definitive analysis.

For both the evaluation of WMH and vascular steal, we utilized a semi-quantitative, observer dependent scoring system: either the Fazekas scale for WMH or an adopted model for steal. Alternative approaches for analysis exist, such as quantitative ΔCBF analysis or ROI-based measurements. Such approaches have been utilized by the authors evaluating pharmaceutical reactivity or correlation with angiographic findings [23,24]. In this particular context, the Fazekas score allows the utilization of a well-understood and clinically ubiquitous scoring system. Additionally, the Fazekas score has demonstrated moderate-to-good inter-rater reliability in cross-sectional evaluation [25].

The ability to create subtraction images from ASL CBF maps, which were acquired within the same acquisition, allowed for mapping of augmentation and steal. This provided both qualitative displays of subtle perfusion changes as well as fused overlay on anatomic images (Figure 2c). This allowed for the development of a modified Fazekas score. However, the applicability of this methodology could be limited in other imaging modalities, such as SPECT, DSC perfusion MRI, or CT. The generalizability of these methods in various modalities would warrant future research and may be institutionally dependent. Additionally, while the Fazekas score has demonstrated good inter-rater reliability, such inter-rater reliability for the modified scoring system is not yet assessed.

We utilized the ASCVD score as a surrogate for vascular health. In our study, we found no statistical significance between the ASCVD score and the amount of cerebrovascular steal or T2 hyperintensities. However, there were several limitations in correlative analysis. Primarily, the calculation utilized in ASCVD calculation is optimized to predict the 10-year risk of an atherosclerotic cardiovascular event, which may have similar etiologies to cerebrovascular events but different weighting of individual variables. Also, variables specific to cerebrovascular disease would not be included in this analysis. Additionally, the data acquisition was retrospective in nature and therefore not standardized. The ASCVD risk assessment is recommended to be performed prior to starting therapy with subsequent recalculations throughout the course of treatment [26]. However, patients obtained an acetazolamide challenge MRI throughout various stages of their cardiovascular risk factor management. Lastly, several patients were not able to be included in the evaluation due to the age restriction of 40–75 or missing clinical data. These limitations may have resulted in nonsignificant findings and a more targeted scoring system of cerebrovascular health, which would be a beneficial topic of future research.

Additionally, technical limitations include the utilization of 8-channel coils for MR imaging. Using more recently available higher channel coils would produce better signal-to-noise ratio and spatial resolution. This could improve statistical significance through the detection of smaller perfusion changes and may allow for a more specific anatomic delineation of white matter tracts. Also, the reproducibility of augmentation outcomes under repeated acetazolamide evaluations was not assessed, and thus, reproducibility cannot be established.

## 5. Conclusions

We conclude that, within the cerebral hemisphere, there is a significant steal phenomenon which shunts blood away from the cerebral white matter structures in the setting of increased cerebrovascular demand. This occurs even in situations of preserved cerebrovascular inflow. Furthermore, the statistically significant relationship between the severity of steal and T2 hyperintensities further implies that this steal contributes, to some meaningful degree, to the etiology of chronic microvascular small vessel disease. Further research is needed to delineate exactly how hemodynamic fluctuations can contribute to the etiologies of leukoaraiosis formation, and its long-term clinical impact.

## Figures and Tables

**Table 1:** Patient demographics.

Demographic	Count or Mean
Age (St. Dev.)	52.52 (17.41)
Sex	
Male	22
Female	16
Stenosis Laterality	
Left	16
Right	22
Diagnosis	
Moya Moya *	5
Intracranial ICA stenosis **	5
MCA Stenosis	8
Cervical ICA stenosis	5

* Clinical diagnosis of Moya Moya disease or syndrome. ** Intracranial ICA stenosis is not listed as Moya Moya disease in the electronic medical record.

**Table 2:** Patient demographics for ASCVD-10 regression analysis.

Variable	Mean (St. Dev.)	Count (Percent)
Age (years)	57.6 (10.2)	
Total Cholesterol (mg/dL)	170.2 (43.9)	
HDL (mg/dL)	41.2 (12.5)	
Systolic BP (mmHg)	145.2 (32.9)	
ASCVD-10 Score (%)	20.7 (15.4)	
Sex		
Male		17 (63.0%)
Female		10 (37.0%)
Race		
Black or African American		6 (22.2%)
White		19 (70.4%)
Other		2 (7.4%)
History of Diabetes		
Yes		9 (33.3%)
No		18 (66.7%)
History of Smoking		
Yes		21 (77.8%)
No		6 (22.2%)
Antihypertensive Medications		
Taking		13 (48.1%)
Not Taking		14 (51.9%)
Stenosis or Occlusion Laterality		
Right		10 (37.0%)
Left		17 (63.0%)

HDL, high-density lipoprotein; BP, blood pressure; ASCVD-10, Atherosclerotic Cardiovascular Disease Risk Score, 10-year risk of cardiovascular adverse events.

**Table 3:** Fazekas and steal grade.

	Ipsilateral/FLH	Contralateral/NFLH
Fazekas Grade		
0	2	10
1	6	20
2	15	8
3	15	0
Steal Grade		
0	1	4
1	4	15
2	26	18
3	7	1

FLH, flow-limited hemisphere; NFLH, non-flow-limited hemisphere.

**Table 4:** Symmetric measures for correlation between non-flow-limited hemisphere steal and non-flow-limited hemisphere T2 hyperintensities and between ipsilateral T2 hyperintensities and contralateral T2 hyperintensities.

Parameter	Spearman Correlation	Pearson’s R	Cases (n)
NFLH Steal and T2 Hyperintensities			
Value	0.48	0.48	38
Asymp. Std. Error	0.15	0.16	
Approx. T	3.32	3.25	
Ipsilateral and Contralateral T2 Hyperintensities			
Value	0.27	0.32	38
Asymp. Std. Error	0.15	0.14	
Approx. T	1.68	2.04	

**Table 5:** Test statistics for the severity of steal and occlusion location of stenosis.

Chi-Square	Ipsilateral T2	Contralateral T2	Ipsilateral Steal	Contralateral Steal
*X*	0.63	0.53	1.07	0.05
*df*	2	2	2	2
*p*	0.731	0.768	0.587	0.976

**Table 6:** Ordinal logistic regression of steal and overall vascular health.

Variable	Estimate (95% CI)	*p*	ASCVD-10 *p*
Ipsilateral Steal	−0.011	0.651	0.647
Ipsilateral T2	−0.0005	0.984	0.983
Contralateral Steal	0.014	0.558	0.567
Contralateral T2	0.008	0.704	0.723

## Data Availability

The raw data supporting the conclusions of this article will be made available by the authors on request.

## Abbreviations

The following abbreviations are used in this manuscript:

CBF	Cerebral blood flow
AZM	Acetazolamide
NFLH	Non-flow-limited hemisphere
FLH	Flow-limited hemisphere
STROBE	Strengthening the Reporting of Observational Studies in Epidemiology
CVR	Cerebrovascular reserve
ASL	Arterial spin labeling
DSA	Digital subtraction angiography
ICA	Internal Carotid Artery
